# Cooccurrence of Myasthenia Gravis and Epilepsy: A Case Report

**DOI:** 10.7759/cureus.62601

**Published:** 2024-06-18

**Authors:** Intissar Barbouch, Bouchra Ennar, Kaoutar Chhita, Yassine Mebrouk

**Affiliations:** 1 Department of Neurology, Mohammed Vl University Hospital, Faculty of Medicine and Pharmacy, Mohammed First University, Oujda, MAR

**Keywords:** seizure, partial epilepsy, epilepsy, myasthenia, myasthenia gravis (mg)

## Abstract

Myasthenia gravis (MG) is an autoimmune disease characterized by weakness and rapid fatigue of the voluntary muscles. Epilepsy is a neurological disorder marked by recurrent, unprovoked seizures. We present a case of a 34-year-old woman with idiopathic generalized epilepsy who developed MG at 33. While the relationship between MG and epilepsy remains unclear, it is known that antiepileptic drugs can exacerbate MG. The rarity of this association suggests a need for cautious selection of antiepileptic treatments to avoid worsening either condition.

## Introduction

Myasthenia gravis (MG) is recognized as the most prevalent disorder affecting the neuromuscular junction, characterized by a B-cell-mediated autoimmune response. This immune reaction targets essential components such as the acetylcholine receptor (AChR), muscle-specific kinase, lipoprotein receptor-related protein 4, or agrin within the postsynaptic membrane [1]. As a result, patients with MG experience muscle weakness, particularly in the ocular, bulbar, respiratory, axial, and limb muscles, which can worsen with exertion [2]. MG has a reported worldwide prevalence of 40-180 per million people and an annual incidence of 4-12 per million people [1].

Epilepsy, a neurological condition affecting approximately 0.5% to 1.0% of the global population, is often managed with therapies targeting symptom control rather than the underlying cause [3]. However, there is growing recognition of the importance of understanding the root causes of epilepsy to develop more effective treatment strategies. Notably, there is an emerging understanding of the relationship between epilepsy and autoimmune diseases. Specific autoimmune causes, often associated with the presence of autoantibodies, have been identified in a subset of epileptic disorders that were previously considered idiopathic [3,4]. This suggests a potential overlap between autoimmune mechanisms and epileptogenesis, highlighting the need for further research into the underlying pathophysiological mechanisms of epilepsy and its potential autoimmune components [4].

## Case presentation

A 34-year-old woman with a known history of epilepsy presented with partial seizures described as a sensation of discomfort and epigastric heaviness associated with palpitations, followed by secondary generalization. She had been on carbamazepine 1200 mg/day for five years. She was admitted to our neurology department for a fluctuating symptomatology evolving over the past few months, characterized by generalized muscle fatigability associated with drooping of the upper eyelids and double vision, which were more pronounced after physical effort and at the end of the day. Occasionally, she also experienced speech difficulties and a nasal voice without any respiratory or swallowing issues. On neurological examination, her cognitive function, cranial nerves, motor strength, and sensory systems were all assessed and found to be within normal limits. Deep tendon reflexes were normal, and there were no signs of cerebellar dysfunction or abnormal involuntary movements. Despite her reported symptoms, there was no clinical evidence of focal neurological deficits during the examination.

**Figure 1:**
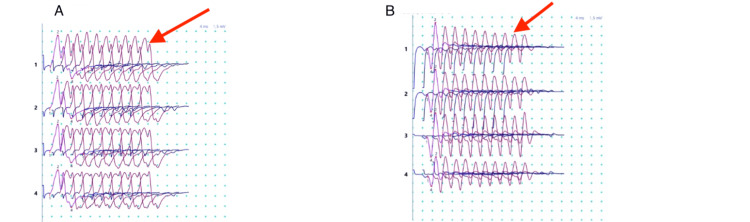
Electromyography showed 10% decrement upon stimulation at 3 Hz in the radial-anconeus muscle-nerve pair, both on the right and left sides (red arrows). (A): Right. (B): Left

The diagnosis of generalized MG was established based on the highly suggestive clinical presentation, positive anti-AChR antibodies, and the presence of a decremental response on repetitive nerve stimulation during electromyography (Figure 1).

**Figure 2:**
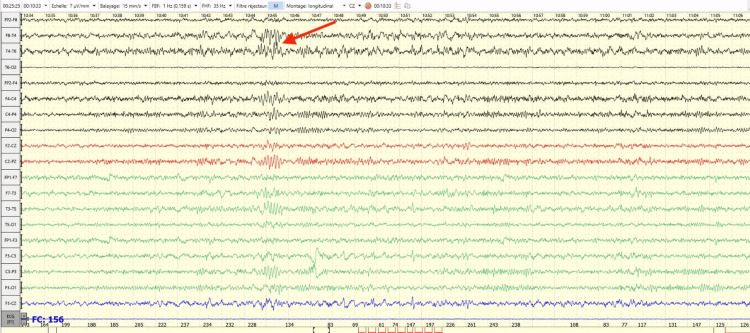
Electroencephalogram revealed bilateral temporal sharp waves (red arrow), suggestive of focal epilepsy

She underwent an electroencephalogram (EEG) (Figure 2) that revealed paroxysmal abnormalities in the right temporal region spreading to both hemispheres, as well as a brain MRI and thoracic CT scan, both of which showed no abnormalities. The decision was to put the patient on anticholinesterase treatment with 240 mg of pyridostigmine per day divided into four doses, along with an adjustment of her antiepileptic treatment, replacing carbamazepine with lamotrigine, which resulted in near-total control of her epileptic seizures.

After seven years of follow-up, the patient remains stable on a combination of lamotrigine and levetiracetam for epilepsy, and her MG is well-controlled with a low dose of pyridostigmine daily.

## Discussion

In patients with MG, there is an increased prevalence of psychiatric disorders, multiple sclerosis, EEG changes, and epilepsy [4].

Two hypotheses have been proposed regarding the association between MG and epilepsy. Firstly, respiratory muscle weakness in myasthenic individuals can lead to hypoventilation, hypoxia, and sleep apnea, thereby causing EEG changes such as mild diffuse slowing.

Secondly, certain antibodies, such as the anti-NMDAR antibody, have been implicated in increasing the risk of seizures and are often associated with autoimmune disorders like MG. These antibodies, including anti-NMDAR, can reduce the density of NMDA receptors, thus impairing their synaptic function. Given the autoimmune nature of MG, there may be an association with other autoimmune diseases and epileptogenic antibodies, such as anti-NMDAR, which are found in a subset of epileptic disorders [5-7].

While these hypotheses outline how generalized MG could lead to epilepsy, it is unlikely that epilepsy directly triggered MG in our patient.

We also discuss the potential influence of prolonged antiepileptic therapy on the development of myasthenic disease [8]. In our case, prolonged exposure to carbamazepine appears to be the most plausible mechanism.

Carbamazepine, commonly used in the treatment of partial epilepsy, may elicit an autoimmune response in susceptible individuals, particularly those with HLA B8. Previous reports have linked carbamazepine to drug-induced lupus syndromes, characterized by positive antinuclear antibodies and double-stranded DNA antibodies. It is conceivable that carbamazepine could similarly induce antibodies against acetylcholine receptors, thereby precipitating myasthenic symptoms. Drug-induced neuromuscular blockade resulting in MG is well-documented, and carbamazepine may expedite the manifestation of underlying MG, similar to phenytoin and lithium salts [8].

## Conclusions

In our study, we report a unique case that establishes a connection between partial epilepsy, prolonged carbamazepine usage, and the subsequent development of MG. While the observed association may initially seem coincidental, it prompts consideration of clinical myasthenic syndrome as a potential complication arising from carbamazepine therapy. This finding underscores the importance of maintaining clinical vigilance among clinicians, especially when managing patients undergoing extended antiepileptic treatment, particularly with carbamazepine. By remaining attentive to the emergence of neuromuscular symptoms, healthcare providers can ensure prompt identification and effective management of potential myasthenic manifestations, thereby enhancing patient care and outcomes.
